# Do social networks affect the use of residential aged care among older Australians?

**DOI:** 10.1186/1471-2318-7-24

**Published:** 2007-10-04

**Authors:** Lynne C Giles, Gary FV Glonek, Mary A Luszcz, Gary R Andrews

**Affiliations:** 1 Department of Rehabilitation and Aged Care, Flinders University, GPO Box 2100, Adelaide 5001, Australia; 2 Discipline of Statistics, The University of Adelaide, Adelaide 5005, Australia; 3 School of Psychology, Flinders University, GPO Box 2100, Adelaide 5001, Australia; 4 Centre for Ageing Studies, Flinders University, GPO Box 2100, Adelaide 5001, Australia

## Abstract

**Background:**

Older people's social networks with family and friends can affect residential aged care use. It remains unclear if there are differences in the effects of specific (with children, other relatives, friends and confidants) and total social networks upon use of low-level residential care and nursing homes.

**Methods:**

Data were drawn from the Australian Longitudinal Study of Ageing. Six waves of data from 1477 people aged ≥ 70 collected over nine years of follow-up were used. Multinomial logistic regressions of the effects of specific and total social networks on residential care use were carried out. Propensity scores were used in the analyses to adjust for differences in participant's health, demographic and lifestyle characteristics with respect to social networks.

**Results:**

Higher scores for confidant networks were protective against nursing home use (odds ratio [OR] upper versus lower tertile of confidant networks = 0.50; 95%CI 0.33–0.75). Similarly, a significant effect of upper versus lower total network tertile on nursing home use was observed (OR = 0.62; 95%CI 0.43–0.90). Evidence of an effect of children networks on nursing home use was equivocal. Nursing home use was not predicted by other relatives or friends social networks. Use of lower-level residential care was unrelated to social networks of any type. Social networks of any type did not have a significant effect upon low-level residential care use.

**Discussion:**

Better confidant and total social networks predict nursing home use in a large cohort of older Australians. Policy needs to reflect the importance of these particular relationships in considering where older people want to live in the later years of life.

## Background

At any point in time in Australia, around one in ten older people have left their home to receive either respite or permanent care in a residential care facility [1]. The Australian aged care system is a tiered system that comprises both community and residential aged care places. Residential aged care may be provided as either 'high-level' or 'low-level' care, depending on clients' needs. In the Australian aged care system, high-level care is equivalent to nursing home care in other countries, and reflects high levels of medical and personal care needs. Low-level residential care (also referred to as 'hostel care' in the Australian system) provides help and housing to older people who do not need continual, high level access to nursing care but have physical, medical, psychological or social care needs that cannot be met through living in the community [2].

Both high-level and low-level residential aged care services are predominantly funded and regulated by the Australian Government [1]. Currently a total of 88 residential aged care places per 1,000 people aged 70 years or more is provided in the Australian aged care system [1]. Religious and charitable organizations deliver the majority of residential aged care services in Australia, although publicly listed companies and small community-based organizations also deliver residential aged care services to a significant number of older people.

Unlike some other countries with specific taxation levies or social insurance programs, the Australian Government funded services are financed from general taxation revenue and user contributions [1]. From both individual and societal perspectives, there are high personal and financial costs associated with admission to residential care [3]. For the Australian Government, the costs of supplying aged care services are forecast to increase from $7.8 billion in 2002–2003 to $106.8 billion in 2042–2043 [4].

A substantial body of U.S. dominated research has identified factors including increasing age, female gender, lack of a marital partner, greater income, better education, lack of home ownership, more comorbid conditions, poorer self-rated health, prior nursing home use, more physical disability, and poorer cognitive status as significant predictors of residential care use, as reviewed recently [5,6]. The findings have been drawn from both cross-sectional and longitudinal studies with follow-up time that varied between one and twenty years, with the median length of follow-up equal to three years.

Social networks with family and friends may be particularly important in providing care to older people, and may thereby delay or prevent admissions to residential care [7-12]. However, few studies have distinguished between social networks with family and those with friends and separately examined their effects on use of residential care. Among those studies that have made this distinction, findings are conflicting. For example, Wolinksy et al. [13] reported non-kin networks were protective but kin networks were not, whereas Freedman [9] demonstrated networks with daughters and siblings, but not sons, were protective against nursing home use. The meta-analysis by Gaugler et al. [5] demonstrated that a greater number of children was protective against nursing home admission, although it is worth noting that only three studies were available for pooling in their analysis of the effects of children, reflecting the paucity of evidence in this area.

It is also likely that social networks are themselves related to some of the factors that have been demonstrated as predictors of residential care use. Therefore it is difficult to make a clear interpretation of the effects of specific and total social networks on residential care based on the existing literature.

Surprisingly little is known about the factors that predict residential care use in Australia. Two recent Australian studies have examined some health, function and lifestyle risk factors for entry to nursing homes. McCallum et al. [13] showed increasing age, incontinence, impaired respiratory flow, more disability, depression, male gender and lower alcohol intake were associated with nursing home use over a 14 year period. Wang et al. [14] found older age, poorer self-rated health, walking disability, current smoking, and lower alcohol consumption were risk factors for admission to nursing homes. However, both studies were set in narrowly defined geographic regions in the same Australian state and were focussed on cardiovascular and ophthalmologic factors respectively. Thus the generalisability of the findings to the wider Australian population remains unclear. Furthermore, social networks and low-level residential care were not considered in these studies. These important gaps in knowledge are addressed in the research reported here.

The primary aim of the present study was to consider the effects of specific types of networks (i.e. those with children, other relatives, friends, confidants, and total social networks) upon use of both low-level residential care and nursing homes in a large sample of older Australians, adjusting for the effects of a wide range of health, function and lifestyle variables. A secondary aim was to examine the effects of putative risk factors on use of nursing homes to add to the knowledge gained from the two previous Australian studies.

## Methods

### Sample

This study uses data from the Australian Longitudinal Study of Ageing (ALSA), a large epidemiological study which aims to increase our understanding of how social, biomedical, behavioural, economic and environmental factors are associated with age-related changes in the health and social well-being of older persons. The study has been described in detail elsewhere [15,16]. In brief, ALSA began in 1992 and is continuing with survivors of the original cohort. The primary sample for ALSA was randomly selected from the South Australian Electoral Roll, and was stratified by Local Government Area (LGA), gender, and five year age groups from 70–74 years through to 85 years and over. Older males aged 85 years or more were deliberately over-sampled to provide sufficient numbers of males for longitudinal follow-up. Persons identified through the Electoral Roll were defined as eligible for the study if they were resident in the Adelaide Statistical Division and were aged 70 years or more on 31 December 1992. Both community-dwelling and people living in residential care were eligible to take part in ALSA, although the majority of participants (91%) were living in the community at baseline interview. A total of 1477 eligible people took part in wave 1 (56% response rate).

Ethical approval for the study was granted by the relevant institutional ethics committee, and each study participant provided written informed consent.

### Data collection and measures

Eight waves of data have been collected from participants between 1992 and 2005, with fieldwork for a ninth wave due to commence in late 2007. In the present article, data from the first six waves were analysed. Waves 1 to 4 were annual interviews that began in 1992, and consenting participants were re-interviewed in 1993, 1994 and 1995. Wave 5 occurred in 1998, and wave 6 was conducted in 2000–2001. Waves 1, 3 and 6 involved detailed personal interviews that covered demographic, medical, psychological, social and economic areas of participants' lives. As well, clinical assessments of participants were carried out in these waves. The clinical examination included anthropometric, neuropsychological, physical performance, balance, and gait measures. Both the interview and clinical assessment were carried out in the participant's usual place of residence. Waves 2, 4 and 5 each consisted of a brief telephone interview that concentrated mainly on health and lifestyle.

### Residential care use

At each interview, participants were classified as living in the *community*, *low-level residential care*, or *nursing home*. For waves 2 through 6, participants were classified as *missing *if they refused an interview or were untraceable. Ongoing searches of the database of official death certificates identified the participants who had *died*, and this approach has been validated previously by the authors [17].

Variables that summarized any use of low-level care or nursing homes over the nine-year study period were also created. For low-level care use, participants were classified as *never using*, *using low-level care*, *already in nursing home at wave 1*, or *missing*. Participants who died without known use of residential care were classified as *never using*. An analogous variable was created to reflect nursing home use. Participants' status was classified as missing if use of the relevant residential care could not be ascertained from the available data.

### Social networks

Adapting the approach of Glass and colleagues [18], confirmatory factor analyses of the wave 1 data were used to develop measures of social networks with children, other relatives, friends, confidants and total social networks. The derivation of the social network variables has been reported previously [19]. Briefly, the children network combined information on the number and proximity of children, and frequency of personal and phone contact with children. The relatives network was composed of the number of relatives, apart from spouse and children, the participant felt close to, and the frequency of personal and phone contact with such relatives. Similarly, the friends network captured the number of close friends, and frequency of personal and phone contact with friends. The confidant network reflected the existence of confidants and whether the confidant was a spouse. A total social network score was calculated as the sum of the children, relatives, friends, and confidant scores. Social network variables were then categorised according to their tertiles, and the tertile classification for each social network was used in further analyses.

### Propensity scores

A range of personal, health, and lifestyle variables were considered important covariates (see Table 1). Geographic area (with 24 levels that designate locality) was also included as a covariate, but is excluded from Table 1 for space considerations. There were many covariates and their distributions were unbalanced among the social network categories, in that participants in different social network categories (i.e. low, medium or high) tended to have different demographic, health and lifestyle characteristics.

**Table 1:** Summary of baseline covariates and association with any nursing home use over study period

**Variable**	**Classification**	**n (%)**	**Odds ratio (95%CI) (n = 909)**^a^
Age group	70–74	379 (25.7%)	Referent
	75–79	352 (23.8%)	4.2 (1.7 – 10.3)
	80–84	341 (23.1%)	3.7 (1.5 – 9.0)
	85+	405 (27.4%)	4.1 (1.7 – 9.8)
Gender	Male	928 (62.8%)	Referent
	Female	549 (32.2%)	0.7 (0.4 – 1.2)
Education	Left school >14 yrs	633 (42.9%)	Referent
	Left school ≤14 yrs	830 (56.2%)	1.0 (0.6 – 1.5)
	Missing	14 (0.9%)	
Marital status	Married/de facto	771 (52.2%)	Referent
	Widowed	586 (40.0%)	1.2 (0.5 – 2.8)
	Single	120 (8.1%)	1.4 (0.8 – 2.4)
Household income	>$AUD12,000	779 (52.7%)	Referent
	≤$AUD12,000	590 (39.9%)	2.0 (1.2 – 3.2)
	Missing	108 (7.3%)	
Home ownership	Owns home	1038 (71.0%)	Referent
	Renting	242 (16.4%)	0.8 (0.5 – 1.5)
	Other	50 (3.4%)	0.4 (0.1 – 1.8)
	In residential care	137 (9.3%)	4.8 (2.5 – 9.0)
Number of chronic conditions^b^	0	264 (17.9%)	Referent
	1	494 (33.4%)	1.3 (0.7 – 2.4)
	2	421 (28.5%)	0.9 (0.5 – 1.8)
	3+	298 (20.2%)	0.6 (0.3 – 1.2)
Self-rated health	Excellent/very good	563 (38.1%)	Referent
	Good	440 (29.8%)	1.2 (0.7 – 2.0)
	Fair/poor	469 (31.8%)	1.4 (0.8 – 2.5)
	Missing	5 (0.3%)	
Hearing difficulty	No	726 (49.2%)	Referent
	Yes	746 (50.5%)	1.5 (1.0 – 2.3)
	Missing	5 (0.3%)	
Difficulty with (corrected) vision [43]	No	1035 (70.1%)	Referent
	Yes	375 (25.4%)	1.3 (0.8 – 2.1)
	Missing	67 (4.5%)	
Mobility disability [44,45]	No disability	949 (64.3%)	Referent
	Disability	506 (34.3%)	1.5 (0.9 – 2.4)
	Missing	22 (1.4%)	
Depressive symptoms CES-D [46]	<17/60	1181 (80.0%)	Referent
	≥17/60	219 (14.8%)	1.2 (0.7 – 2.1)
	Missing	77 (5.2%)	
Cognitive function [40, 47]	>16/21	1221 (82.7%)	Referent
	≤16/21	219 (14.8%)	1.6 (0.9 – 2.7)
	Missing	37 (2.5%)	
Alcohol consumption (AUDIT) [48]	<8/10	1401 (94.9%)	Referent
	≥8/10	65 (4.4%)	1.0 (0.3 – 3.0)
	Missing	11 (0.7%)	
Exercise status [16, 49]	Exerciser	794 (53.8%)	Referent
	Sedentary	663 (44.9%)	1.0 (0.7 – 1.6)
	Missing	20 (1.4%)	
Smoking status	Never	661 (44.8%)	Referent
	Former	667 (45.2%)	0.6 (0.4 – 1.0)
	Current	123 (8.3%)	0.5 (0.2 – 1.2)
	Missing	16 (1.1%)	

a: analysis based on data from 909 participants with complete information on both nursing home use (n = 1078) and risk factors (n = 1243)

b: self-reported ever suffering from arthritis, cancer, chronic bronchitis or emphysema, diabetes, fractured hip, heart attack, heart condition, hypertension, osteoporosis, stroke

In randomized controlled trials, group assignment is, by definition, randomly allocated and so the differences in observed covariates between treatment groups should be minimized. However, in observational studies such as ALSA, there is no manipulation of 'treatment' assignment, and so there is the potential for large differences between observed covariates in the different treatment groups. Ignoring these differences could potentially lead to biased estimates of treatment effects. Traditional methods of adjusting for observed covariates in analyses, such as matching or stratification, may be difficult to use if there are a large number of covariates. Regression adjustment can also be problematic. Missing values in one or more covariates for an individual will result in all data for that individual being dropped from a regression analysis unless estimation of the missing covariate values is carried out. Another potential problem in regression adjustment is that finding and fitting an appropriate functional form for each covariate may be difficult.

Propensity scores have been proposed as an alternative method to adjust for a set of covariates [20,21]. Most applications of propensity scores to date have involved simple cross-sectional studies with binary treatments. More recent work [21] that extends the derivation of propensity scores to treatments with multiple categories is applicable in the present study. In our study the 'treatment', social network tertile, has three categories corresponding to the low, mid, or high tertile of the relevant social network score for each of the specific and total social networks.

We turn now to a more formal definition of propensity scores, and first consider their derivation for a binary treatment. Let *Z*_*i *_be an indicator variable of assignment to a treatment for individual *i*, such that Zi={1if treated0if control
 MathType@MTEF@5@5@+=feaafiart1ev1aaatCvAUfKttLearuWrP9MDH5MBPbIqV92AaeXatLxBI9gBaebbnrfifHhDYfgasaacH8akY=wiFfYdH8Gipec8Eeeu0xXdbba9frFj0=OqFfea0dXdd9vqai=hGuQ8kuc9pgc9s8qqaq=dirpe0xb9q8qiLsFr0=vr0=vr0dc8meaabaqaciaacaGaaeqabaqabeGadaaakeaacqWGAbGwdaWgaaWcbaGaemyAaKgabeaakiabg2da9maaceqabaqbaeaabiGaaaqaaiabigdaXaqaaiabbMgaPjabbAgaMjabbccaGiabbsha0jabbkhaYjabbwgaLjabbggaHjabbsha0jabbwgaLjabbsgaKbqaaiabicdaWaqaaiabbMgaPjabbAgaMjabbccaGiabbogaJjabb+gaVjabb6gaUjabbsha0jabbkhaYjabb+gaVjabbYgaSbaaaiaawUhaaaaa@4DA4@.

The propensity score *p*(***x***_*i*_) is defined as the conditional probability of assignment to treatment versus control given a vector of observed covariates ***x***_*i*_. More formally *p*(***x***_*i*_) = Pr(*Z*_*i *_= 1|***X***_*i *_= ***x***_*i*_) under the assumption that, given the ***X***_*i*_, the *Z*_*i *_are independent – that is, Pr⁡(Z1=z1,...,Zn=zn|X1=x1,...,Xn=xn)=∏i=1np(xi)zi{1−p(xi)}1−zi
 MathType@MTEF@5@5@+=feaafiart1ev1aaatCvAUfKttLearuWrP9MDH5MBPbIqV92AaeXatLxBI9gBaebbnrfifHhDYfgasaacH8akY=wiFfYdH8Gipec8Eeeu0xXdbba9frFj0=OqFfea0dXdd9vqai=hGuQ8kuc9pgc9s8qqaq=dirpe0xb9q8qiLsFr0=vr0=vr0dc8meaabaqaciaacaGaaeqabaqabeGadaaakeaacyGGqbaucqGGYbGCcqGGOaakcqWGAbGwdaWgaaWcbaGaeGymaedabeaakiabg2da9iabdQha6naaBaaaleaacqaIXaqmaeqaaOGaeiilaWIaeiOla4IaeiOla4IaeiOla4IaeiilaWIaemOwaO1aaSbaaSqaaiabd6gaUbqabaGccqGH9aqpcqWG6bGEdaWgaaWcbaGaemOBa4gabeaakiabcYha8Hqadiab=HfaynaaBaaaleaacqaIXaqmaeqaaOGaeyypa0Jae8hEaG3aaSbaaSqaaiabigdaXaqabaGccqGGSaalcqGGUaGlcqGGUaGlcqGGUaGlcqGGSaalcqWFybawdaWgaaWcbaGaemOBa4gabeaakiabg2da9iab=Hha4naaBaaaleaacqWGUbGBaeqaaOGaeiykaKIaeyypa0ZaaebCaeaacqWGWbaCcqGGOaakcqWF4baEdaWgaaWcbaGaemyAaKgabeaakiabcMcaPmaaCaaaleqabaGaemOEaO3aaSbaaWqaaiabdMgaPbqabaaaaOGaei4EaSNaeGymaeJaeyOeI0IaemiCaaNaeiikaGIae8hEaG3aaSbaaSqaaiabdMgaPbqabaGccqGGPaqkcqGG9bqFdaahaaWcbeqaaiabigdaXiabgkHiTiabdQha6naaBaaameaacqWGPbqAaeqaaaaaaSqaaiabdMgaPjabg2da9iabigdaXaqaaiabd6gaUbqdcqGHpis1aaaa@7693@. In other words, *p*(***x***_*i*_) is a measure of the probability that an individual would have been treated based on only the individual's covariate information [22]. Propensity scores 'balance' the observed covariates, in that the conditional distribution of ***X***_*i *_given *p*(***x***_*i*_) is the same for individuals, irrespective of whether they receive treatment or control. In other words, *Z*_*i *_and ***X***_*i *_are conditionally independent given *p*(***x***_*i*_). The success of the propensity scores in balancing the covariates can be checked through simple comparisons of the treatment and control groups that adjust for the propensity scores in the analyses [22].

Applications of propensity score adjustment with more than two treatment categories have not been widely reported. For three or more levels of treatment, Joffe and Rosenbaum [21] showed that if the distribution of treatment doses given ***X***_*i *_is accurately described by McCullagh's proportional odds model, then stratifying on *b*(***x***_*i*_) = ***x***_*i*_'***β ***where ***β ***is a *p *× 1 vector of parameters, will balance ***X***_*i *_across several dose groups. More generally, it is possible that the distribution of doses *Z *given a large number of covariates may depend on the covariates through only a small number of linear functions of ***X***, say ***X*G **for some matrix **G**. Then controlling for the several variables in ***X*G **will 'tend to balance the ... variables in ***X***' [21]. For example, if a multinomial logistic regression model was adequate to describe Pr(*Z*_*i *_= *z*|***X***_*i *_= ***x***_*i*_) for some *z *= 0, 1, ..., *c*, then ***X*G **would be an *n *× *c *matrix in which each of the *c *columns defined a propensity score for level *c *of the treatment dose. Because of the linear dependence of the *c*^th ^propensity score on the first *c*-1 propensity scores, analyses would adjust for the first *c*-1 propensity scores.

To obtain propensity scores in the present analysis, an ordinal logistic regression of each of the social network tertiles on the covariates was initially fit for participants with complete covariate data (n = 1243). A pragmatic approach was adopted so that if, for a given participant, an observation was missing for at least one of the covariates, a propensity score was estimated using the subset of covariates with complete data for that participant. In this way, a propensity score was estimated for every participant, not only those participants with complete data for all covariates, and a propensity score was estimated for each pattern of missing covariate observations. Thus for every participant, the propensity scores were estimated using the maximum covariate information available for that participant.

Ordinal logistic models were appropriate for the distribution of the children, relatives, friends and total social network variables given the observed covariates, but not for confidants. For the confidant network variable, a multinomial logistic regression model was used. The resulting conditional probabilities of being in each of the three confidant network categories defined the three propensity scores for the confidant social network [23,24]. Because of the linear dependence of the third propensity score on the other two, only the first two propensity scores were included in subsequent analyses of the effect of the confidant social network upon use of residential care.

Rosenbaum and Rubin [20], based on work by Cochran [25], stated that five strata based on the propensity score would remove over 90% of the bias in each of the covariates. Thus when an ordinal logistic model was used, participants were sub-classified into quintiles based on the propensity scores. When the multinomial logistic model was used, participants were sub-classified into nine strata based on the joint distribution of their first two propensity scores. The propensity score strata for each participant was included in all analyses.

The balance of the covariates in each of the propensity score strata in the present study was examined by chi-square tests of association of each covariate with each of the categorised social network variables [23]. A total of 25 out of 484 comparisons for balance status of the covariates (5%) were statistically significant at P < 0.05. This indicated that the propensity score method produced balance in the observed covariates similar to that which would be expected by randomization of these covariates across the social network tertiles. On this basis, it was determined that the propensity scores provided an adequate adjustment.

### Statistical analysis

Several analyses of place of residence were conducted. First, the effects of the putative risk factors on any nursing home use over the study period were examined to enable comparison with previous Australian studies [13,14]. An unordered, multinomial, multiple logistic regression model that included the factors shown in Table 1 and geographic area was fit.

Second, separate logistic regression models of i) any low-level care use and ii) nursing home use across the study period on each social network variable were fit, adjusting for propensity score strata. Sensitivity analyses, in which missing values were imputed as never used (most conservative) or all used (most extreme), were conducted to compare the effects of different assumptions regarding missing values with the analyses that used only available data.

Finally, the place of residence across the six study waves was longitudinally analysed, with response categories of community, low-level care, nursing home, or dead possible at each wave. A separate multinomial logistic regression model of place of residence at waves 2–6 on each social network was fit, adjusting for propensity score strata, study wave, and place of residence at the previous wave. The Huber-White robust variance estimator was used to account for the repeated observations (n ≤ 5 observations corresponding to waves 2–6) from each participant [26,27].

## Results

Table 1 summarises the baseline characteristics of the 1477 participants. The average age at selection was 79.8 years (SD = 6.9), and close to two-thirds of the sample were male. More than half of the participants had left school before the age of 15 years, and approximately half of the sample was married/partnered. Participants most commonly had one morbid condition, and 15% of participants showed some signs of cognitive deficits. More than half of the participants were former or current smokers, and almost half of the participants were sedentary. Also shown in Table 1 are odds ratios that describe the association of any nursing home use with each of the risk factors. These results are described later in this section.

Across the entire study period, a total of 778 participants (53%) did not use low-level care, or died without use, and a further 136 participants (9%) were either in a nursing home at wave 1 or moved directly to a nursing home from the community. Low-level care was known to have been used over the study period by 189 participants (13%). Information on use of low-level care could not be ascertained for 374 participants (25%) because they were alive but not interviewed at one or more waves, and thus their use of residential care at the missing wave(s) could not be determined.

Over the nine years of the study, 883 participants (60%) never used a nursing home or died without use, while 195 participants (13%) used a nursing home. Nursing home use could not be ascertained for the remaining 399 participants (27%), for the same reason as those with missing low-level care information.

The place of residence at each wave is shown in Table 2. The percentage of the surviving cohort living in the community decreased over the nine years from 91% at wave 1 to 82% at wave 6. Between 6% and 8% of participants lived in low-level care at each of the waves. The proportion of participants who were resident in nursing homes increased over time, from 3% at wave 1 to 12% at wave 6.

**Table 2:** Place of residence at each wave

**Year**	**Wave**	**Community**	**Low-level care**	**Nursing Home**	**Missing**	**Dead**
**1992**	**1 (n)**	1,340	92	45	0	0
	**(% all)**	91	6	3	0	0
**1993**	**2 (n)**	1,126	83	51	137	80
	**(% all)**	76	6	4	9	5
	**(% alive)**	89	7	4		
**1994**	**3 (n)**	1,030	80	61	113	193
	**(% all)**	70	5	4	8	13
	**(% alive)**	88	7	5		
**1995**	**4 (n)**	900	74	62	150	291
	**(% all)**	61	5	4	10	20
	**(% alive)**	87	7	6		
**1998**	**5 (n)**	646	64	51	210	506
	**(% all)**	44	4	4	14	34
	**(% alive)**	85	8	7		
**2000**	**6 (n)**	412	28	60	215	762
	**(% all)**	28	2	4	14	52
	**(% alive)**	82	6	12		

Shown are number and per cent of all participants (% all) and surviving participants (% alive).

A total of 909 participants had complete data concerning any nursing home use across the nine-year study period and the putative risk factors. As summarized in Table 1, age group, lower household income, lack of home ownership and hearing difficulty were significant risk factors for nursing home use over the study period.

The effects of social networks on use of low-level care and nursing home use were then explicitly considered. As shown in Table 3, better social networks with children, confidants and total social networks appeared protective against any nursing home use across the study period, after adjusting for propensity score strata. However, only the upper tertile of children networks in comparison to the lower tertile had a significant effect on any nursing home use. Moreover, there was no evidence of a gradient of the effect of children networks on any nursing home use.

**Table 3:** Summary of effect of social networks upon any nursing home use and any low-level care use

	**Low-level care**^a^		**Nursing Home**^b^	
	**OR**	**95% CI**	**OR**	**95% CI**
**Any use over study period**				
Children				
Mid tertile	1.44	0.97 – 2.15	1.02	0.69 – 1.50
Upper tertile	0.97	0.64 – 1.46	0.60	0.40 – 0.90
Overall χ22 MathType@MTEF@5@5@+=feaafiart1ev1aaatCvAUfKttLearuWrP9MDH5MBPbIqV92AaeXatLxBI9gBaebbnrfifHhDYfgasaacH8akY=wiFfYdH8Gipec8Eeeu0xXdbba9frFj0=OqFfea0dXdd9vqai=hGuQ8kuc9pgc9s8qqaq=dirpe0xb9q8qiLsFr0=vr0=vr0dc8meaabaqaciaacaGaaeqabaqabeGadaaakeaacqaHhpWydaqhaaWcbaGaeGOmaidabaGaeGOmaidaaaaa@3074@^c^	4.81		8.60*	
Relatives				
Mid tertile	0.98	0.66 – 1.44	0.81	0.56 – 1.18
Upper tertile	1.00	0.65 – 1.53	0.76	0.50 – 1.17
Overall χ22 MathType@MTEF@5@5@+=feaafiart1ev1aaatCvAUfKttLearuWrP9MDH5MBPbIqV92AaeXatLxBI9gBaebbnrfifHhDYfgasaacH8akY=wiFfYdH8Gipec8Eeeu0xXdbba9frFj0=OqFfea0dXdd9vqai=hGuQ8kuc9pgc9s8qqaq=dirpe0xb9q8qiLsFr0=vr0=vr0dc8meaabaqaciaacaGaaeqabaqabeGadaaakeaacqaHhpWydaqhaaWcbaGaeGOmaidabaGaeGOmaidaaaaa@3074@	0.02		1.81	
Friends				
Mid tertile	1.05	0.71 – 1.55	0.78	0.54 – 1.14
Upper tertile	1.29	0.85 – 1.95	0.70	0.46 – 1.06
Overall χ22 MathType@MTEF@5@5@+=feaafiart1ev1aaatCvAUfKttLearuWrP9MDH5MBPbIqV92AaeXatLxBI9gBaebbnrfifHhDYfgasaacH8akY=wiFfYdH8Gipec8Eeeu0xXdbba9frFj0=OqFfea0dXdd9vqai=hGuQ8kuc9pgc9s8qqaq=dirpe0xb9q8qiLsFr0=vr0=vr0dc8meaabaqaciaacaGaaeqabaqabeGadaaakeaacqaHhpWydaqhaaWcbaGaeGOmaidabaGaeGOmaidaaaaa@3074@	1.56		3.13	
Confidants				
Mid tertile	1.53	1.04 – 2.24	0.67	0.46 – 0.97
Upper tertile	1.10	0.67 – 1.79	0.49	0.31 – 0.77
Overall χ22 MathType@MTEF@5@5@+=feaafiart1ev1aaatCvAUfKttLearuWrP9MDH5MBPbIqV92AaeXatLxBI9gBaebbnrfifHhDYfgasaacH8akY=wiFfYdH8Gipec8Eeeu0xXdbba9frFj0=OqFfea0dXdd9vqai=hGuQ8kuc9pgc9s8qqaq=dirpe0xb9q8qiLsFr0=vr0=vr0dc8meaabaqaciaacaGaaeqabaqabeGadaaakeaacqaHhpWydaqhaaWcbaGaeGOmaidabaGaeGOmaidaaaaa@3074@	5.16		10.75*	
Total				
Mid tertile	1.49	1.01 – 2.21	0.55	0.37 – 0.81
Upper tertile	1.25	0.80 – 1.95	0.54	0.35 – 0.83
Overall χ22 MathType@MTEF@5@5@+=feaafiart1ev1aaatCvAUfKttLearuWrP9MDH5MBPbIqV92AaeXatLxBI9gBaebbnrfifHhDYfgasaacH8akY=wiFfYdH8Gipec8Eeeu0xXdbba9frFj0=OqFfea0dXdd9vqai=hGuQ8kuc9pgc9s8qqaq=dirpe0xb9q8qiLsFr0=vr0=vr0dc8meaabaqaciaacaGaaeqabaqabeGadaaakeaacqaHhpWydaqhaaWcbaGaeGOmaidabaGaeGOmaidaaaaa@3074@	4.05		12.21*	

Lower tertile is referent category in all analyses

a: Complete data available for 1103 cases.

b: Complete data available for 1078 cases.

c, *: χ^2^on 2 df;values > 5.99 significant at P < 0.05

There was no significant effect of the specific or total social network variables upon low-level care use. The findings were robust to assumptions regarding the use of residential care by participants with missing data, as the sensitivity analyses did not differ substantively from the main results.

Table 4 summarises the longitudinal analysis of the effect of social networks on place of residence, adjusted for propensity score strata, study wave, and residence at previous wave. As these results show, specific and total social networks did not have a significant effect upon low-level care use. The significant effect observed for the friends network was due to the protective effect of better friend networks upon survival. Higher scores for confidant networks appeared protective against nursing home use (odds ratio [OR] upper versus lower tertile of confidant networks = 0.50; 95%CI 0.33–0.75). Similarly, a significant effect of upper versus lower tertile for the total social network was observed (OR = 0.62; 95%CI 0.43–0.90).

**Table 4:** Summary of effects of social networks upon place of residence across study period

	**Low-level care** **OR**	**95% CI**	**Nursing home** **OR**	**95% CI**	**Dead** **OR**	**95% CI**
**Children**						
Mid tertile	1.03	0.74 – 1.44	1.24	0.89 – 1.72	1.11	0.87 – 1.42
Upper tertile	0.68	0.48 – 0.96	0.85	0.60 – 1.21	1.02	0.79 – 1.31
Overall χ62 MathType@MTEF@5@5@+=feaafiart1ev1aaatCvAUfKttLearuWrP9MDH5MBPbIqV92AaeXatLxBI9gBaebbnrfifHhDYfgasaacH8akY=wiFfYdH8Gipec8Eeeu0xXdbba9frFj0=OqFfea0dXdd9vqai=hGuQ8kuc9pgc9s8qqaq=dirpe0xb9q8qiLsFr0=vr0=vr0dc8meaabaqaciaacaGaaeqabaqabeGadaaakeaacqaHhpWydaqhaaWcbaGaeGOnaydabaGaeGOmaidaaaaa@307C@ = 8.6^a^						
**Relatives**						
Mid tertile	0.93	0.67 – 1.29	0.72	0.53 – 1.00	0.95	0.75 – 1.20
Upper tertile	0.92	0.64 – 1.33	0.74	0.51 – 1.07	1.07	0.82 – 1.38
Overall χ62 MathType@MTEF@5@5@+=feaafiart1ev1aaatCvAUfKttLearuWrP9MDH5MBPbIqV92AaeXatLxBI9gBaebbnrfifHhDYfgasaacH8akY=wiFfYdH8Gipec8Eeeu0xXdbba9frFj0=OqFfea0dXdd9vqai=hGuQ8kuc9pgc9s8qqaq=dirpe0xb9q8qiLsFr0=vr0=vr0dc8meaabaqaciaacaGaaeqabaqabeGadaaakeaacqaHhpWydaqhaaWcbaGaeGOnaydabaGaeGOmaidaaaaa@307C@ = 13.5						
**Friends**						
Mid tertile	1.28	0.91 – 1.81	0.99	0.70 – 1.38	0.92	0.73 – 1.17
Upper tertile	1.22	0.83 – 1.78	0.74	0.52 – 1.07	0.72	0.56 – 0.93
Overall χ62 MathType@MTEF@5@5@+=feaafiart1ev1aaatCvAUfKttLearuWrP9MDH5MBPbIqV92AaeXatLxBI9gBaebbnrfifHhDYfgasaacH8akY=wiFfYdH8Gipec8Eeeu0xXdbba9frFj0=OqFfea0dXdd9vqai=hGuQ8kuc9pgc9s8qqaq=dirpe0xb9q8qiLsFr0=vr0=vr0dc8meaabaqaciaacaGaaeqabaqabeGadaaakeaacqaHhpWydaqhaaWcbaGaeGOnaydabaGaeGOmaidaaaaa@307C@ = 26.4						
**Confidants**						
Mid tertile	0.95	0.69 – 1.32	0.70	0.51 – 0.97	0.84	0.67 – 1.06
Upper tertile	0.86	0.57 – 1.31	0.50	0.33 – 0.75	0.73	0.56 – 0.94
Overall χ62 MathType@MTEF@5@5@+=feaafiart1ev1aaatCvAUfKttLearuWrP9MDH5MBPbIqV92AaeXatLxBI9gBaebbnrfifHhDYfgasaacH8akY=wiFfYdH8Gipec8Eeeu0xXdbba9frFj0=OqFfea0dXdd9vqai=hGuQ8kuc9pgc9s8qqaq=dirpe0xb9q8qiLsFr0=vr0=vr0dc8meaabaqaciaacaGaaeqabaqabeGadaaakeaacqaHhpWydaqhaaWcbaGaeGOnaydabaGaeGOmaidaaaaa@307C@ = 31.0						
**Total**						
Mid tertile	0.77	0.55 – 1.08	0.57	0.40 – 0.81	0.76	0.60 – 0.97
Upper tertile	0.94	0.67 – 1.34	0.62	0.43 – 0.90	0.84	0.65 – 1.08
Overall χ62 MathType@MTEF@5@5@+=feaafiart1ev1aaatCvAUfKttLearuWrP9MDH5MBPbIqV92AaeXatLxBI9gBaebbnrfifHhDYfgasaacH8akY=wiFfYdH8Gipec8Eeeu0xXdbba9frFj0=OqFfea0dXdd9vqai=hGuQ8kuc9pgc9s8qqaq=dirpe0xb9q8qiLsFr0=vr0=vr0dc8meaabaqaciaacaGaaeqabaqabeGadaaakeaacqaHhpWydaqhaaWcbaGaeGOnaydabaGaeGOmaidaaaaa@307C@ = 30.7						

Lower tertile is referent category in all analyses. Community dwelling is referent response category.

a: χ62
 MathType@MTEF@5@5@+=feaafiart1ev1aaatCvAUfKttLearuWrP9MDH5MBPbIqV92AaeXatLxBI9gBaebbnrfifHhDYfgasaacH8akY=wiFfYdH8Gipec8Eeeu0xXdbba9frFj0=OqFfea0dXdd9vqai=hGuQ8kuc9pgc9s8qqaq=dirpe0xb9q8qiLsFr0=vr0=vr0dc8meaabaqaciaacaGaaeqabaqabeGadaaakeaacqaHhpWydaqhaaWcbaGaeGOnaydabaGaeGOmaidaaaaa@307C@ test of effect of social network variable; values > 12.59 significant at P < 0.05.

## Discussion

The effects of specific and total social networks on residential care use were examined over a nine year period, using propensity score methods to adjust for a broad range of covariates. Longitudinal analyses showed better confidant networks and better total social networks were associated with reduced odds of nursing home admission over the course of the study. There was weaker evidence of a significant effect of better children networks on reduced odds of nursing home use, and there was no evidence of an effect of children networks in the longitudinal analyses. There was no significant effect of social networks with other relatives or friends on nursing home use. Furthermore, the results suggested specific and total social networks had little effect on use of low-level residential care over the period of the study.

Increasing age, lower income, and hearing difficulty were shown to be significant risk factors for nursing home use across the course of the study, adding to previous Australian research in this area. The finding regarding hearing difficulty adds more evidence to the need for adequate assessment of sensory impairments at the time of assessment for nursing home placement [14] and ongoing monitoring of auditory acuity. In contrast to visual acuity, hearing difficulties may go unnoticed, as the behavioural consequences may not be immediately obvious in the context of competing demands on staff time and attention. The effect of income on risk of nursing home admission is equivocal in the international literature, with some authors reporting reduced income to increase risk [28,29], while others have shown higher income is a risk factor for nursing home admission [30-33]. The results for income reported here possibly reflect that older Australians with a lower income may not be able to purchase support services to assist them to continue to live in the community, and so are more likely to move to residential care. Furthermore, higher income may be a disincentive to nursing home use in Australia. Substantial entry costs or ongoing costs in addition to the Australian Age Pension can be levied by individual facilities according to means-tested criteria. Issues of equity and access to residential care must remain high on the agenda for Australian aged care policy makers.

Confidant networks were significantly protective against nursing home use in this study, suggesting a close, emotionally supportive relationship with another person is beneficial in preventing or delaying nursing home use. The importance of a confidant to mental and physical health is well known [34-36] but the translation of that effect to a reduction in risk of nursing home use has not been shown previously. Further research is clearly warranted to examine the repeatability of this finding in other settings and countries.

Social networks with relatives and friends had no significant effect on use of residential care. Children networks appeared to have some protective effect against any nursing home use over the study period, but this finding did not extend to the results from the longitudinal analyses. Those with fewer non-kin social supports may have smaller networks of human resources to draw upon for maintenance of community living status [12,37]. Other research has shown significant protection against nursing home use arising from having daughters and siblings [8]. Our research suggests that the core network of confidants, and to a lesser extent children, is more important than other specific networks in delaying or preventing use of nursing homes in Australia. The striking impact of absence of confidants may reflect the consequences of reduced emotional support that permitted continued residence in the community, which would be consistent with Carstensen's socioemotional selectivity theory [38]. Social networks of any of the types considered here had minimal effect upon use of low-level care.

Several limitations to the study must be acknowledged. ALSA non-respondents may have been more socially isolated than participants, although non-response bias has been demonstrated as minimal in other analyses of ALSA data [15,39,40]. The analyses were based on self-reported data and adjusted for covariates that were measured at wave 1. Social networks may have changed over time, but the social networks considered in the present study were based on only wave 1 data. However, total network size has been demonstrated as relatively stable over a long follow-up period in a study of older Dutch people [41]. Furthermore, disentangling the effects of time-varying social networks may be difficult as changes in social networks may be a consequence of changes in place of residence. A final limitation is that date of entry to residential care was not available, and thus residential care use between study waves was not reflected in the data.

Arguably these limitations are balanced by ALSA's strengths, which include the rich baseline data that enabled propensity score adjustment, the broad sample, and the Australian setting, which expands the generalisability of the role of social networks in the use of residential care. The follow-up time in the present study is also notably longer than that of many other international studies in this area. Our results add not only to the general body of knowledge concerning risk factors for residential care use, but also extend the literature to encompass the specific role played by social networks in this important transition. In future research, we will track place of death for study decedents which will reduce the proportion of missing data concerning the use of residential care over time.

ALSA took place against a background of reforms in Australian aged care [42] that may have had an impact on the use of residential care services independent of the risk factors considered here. One of the most significant reforms saw the assessment for entry to low-level and high-level residential aged care merged into one system in 1997. It is important to note that the policy changes did not affect an individual's eligibility for residential aged care, but streamlined the administrative processes concerning assessment criteria. An individual's eligibility for residential aged care is ascertained by Aged Care Assessment Teams (ACAT) against standardized criteria that include functional status, health and living arrangements. The persistent effects of social networks on use of nursing homes over a long period of follow-up and over and above the effects of a range of other variables suggest that an individual's social milieu needs to be reflected more strongly in eligibility criteria, particularly for high-level residential care. The results of the present study also highlight the importance of recognizing that social networks go beyond a simple ascertainment of marital status or number of children. It may be possible to incorporate the findings from the present study in better screening assessments by ACATs for residential care eligibility. Policymakers may need to reconsider whether social relationships have been given adequate weight in the current assessment and entry process.

The effects of social networks on residential care use have not been examined previously in an Australian context. We have shown that social networks with children and total social networks, especially those with confidants, predict nursing home use over nine years in a large cohort of older Australians. Policy needs to reflect the importance of these particular relationships, and incorporate these along with the expectations of future cohorts of older people about where they want to live in later life.

## Competing interests

The author(s) declare that they have no competing interests.

## Authors' contributions

LG conceived of the present study, participated in the design and conduct of the statistical analysis plan and had primary responsibility in drafting the manuscript. GG participated in the design of the study and the statistical analysis plan and participated in drafting of the manuscript. ML participated in the design of the study and in drafting of the manuscript. GA conceived of and directed the Australian Longitudinal Study of Ageing, and participated in the design of the present study.

LG, GG and ML read and approved the final manuscript. GA commented on early drafts of the manuscript but was unable to approve the final version due to his death in May 2006.

## Pre-publication history

The pre-publication history for this paper can be accessed here:


